# Global disparities in neurosurgical literature: a narrative review of underrepresentation of low- and middle-income countries and pathways to research equity

**DOI:** 10.1097/MS9.0000000000005061

**Published:** 2026-07-15

**Authors:** Tirath Patel, Naveed Ahmad, Abbas Hussain, Bisma Faraz, Amna binte Naeem, Urooj Sheraz, Laiba Noor, Saleha Khan, Muhammad Seerat Ali, Hafiza Tooba Siddiqui, Taimur Iqbal, Bhumi Daishik Patel, Nikhilesh Anand

**Affiliations:** a Department of Neurosurgery, Trinity Medical Sciences University School of Medicine, Kingstown, Saint Vincent and the Grenadines; b Department of Neurosurgery, Khyber Medical College, Peshawar, Khyber Pakhtunkhwa, Pakistan; c Department of Neurosurgery, Jinnah Medical and Dental College, Karachi, Pakistan; d Department of Neurosurgery, Karachi Medical and Dental College (KMDC), Karachi, Pakistan; e Department of Neurosurgery, Rawalpindi Medical University, Rawalpindi, Pakistan; f Department of Neurosurgery, Dow Medical College, Karachi, Pakistan; g Department of Neurosurgery, Batterjee Medical College for Sciences and Technology, Jeddah, Saudi Arabia; h Department of Neurosurgery, Quaid-e-Azam Medical College, Bahawalpur, Pakistan; i Department of Neurosurgery, Jinnah Sindh Medical University, Karachi, Pakistan; j Department of Neurosurgery, Windsor University School of Medicine, Cayon, Saint Kitts and Nevis; k Department of Medical Education, University of Texas Rio Grande Valley, Edinburg, TX, USA

**Keywords:** authorship inequity, global neurosurgery, low- and middle-income countries, neurosurgical literature, research disparities

## Abstract

The global neurosurgical literature is dominated by high-income countries, with low- and middle-income countries (LMICs) contributing minimally despite bearing the most significant share of the neurosurgical disease burden. Underrepresentation limits equitable knowledge dissemination and the relevance of guidelines. The objective of this review was to assess neurosurgical publication disparities, identify deterrents to LMIC participation, and outline initiatives that foster equity and inclusion in research. This narrative review synthesized evidence on global disparities in neurosurgical literature through a structured search of PubMed, Scopus, and Google Scholar (2010–2025). Eligible studies examined research output, authorship trends, publication barriers, or equity initiatives. Findings mapped bibliometric patterns, highlighted structural barriers, and synthesized strategies to promote equitable neurosurgical scholarship. More than two-thirds of neurosurgical reviews come from North America and Europe, whereas less than 10% come from Africa and South-East Asia. Barriers to publication include limited funding, high article processing charges, lack of mentorship, and weak institutional infrastructure. LMIC authors are often relegated to middle authorship, which decreases exposure and hinders academic advancement. Case studies from Africa, South Asia, and Latin America highlight progress in expanding the workforce and in training for specific areas, but persistent research inequities remain. Recent initiatives, such as AuthorAID, HINARI, African Neurosurgical Research Collaborative, and open-access waiver programs, have demonstrated measurable improvements in LMIC research output and authorial independence. Significant inequities persist in neurosurgical research. For sustainable solutions, there is an important need for structural reforms: expanded mentorship, equitable funding, inclusive editorial policies, and strengthened regional collaborations that ensure global neurosurgical research reflects diverse contexts and meaningfully contributes to worldwide patient care.

## Introduction

High-quality neurosurgical literature, especially review articles, constitutes the basis of evidence-based practice and policy. Good reviews integrate available evidence, create consensus, and define future research directions^[^1^]^. They inform clinical guidelines that codify care worldwide. Yet, the worldwide body of neurosurgical research remains enormously skewed, with the lion’s share of contributions coming from high-income nations (HICs).

Bibliometric analyses demonstrate the ongoing underrepresentation of authors from low- and middle-income countries (LMICs). More than one-quarter of “global neurosurgery” publications feature zero LMIC-affiliated authors, and fewer than 11% have three or more^[^2^]^. As few as 4–5% of neurosurgical journal publications include any LMIC contribution^[^3^]^, and just 8–9% of open-access publications originate from lower-income countries^[^4,5^]^. Together, these data demonstrate that much of the world’s neurosurgical community remains academically underrepresented^[^4,6^]^.

This imbalance is particularly pronounced in Africa, South Asia, and Latin America. In neurosurgery in Africa, almost all the literature is low-level evidence, with only 4.9% being narrative reviews and 0.8% systematic reviews^[^7^]^. Latin American organizations publish only 4–5% of high-impact neurosurgical articles^[^8^]^, whereas South-East Asian nations continue to experience limited research capacity and resources^[^9^]^. Thus, LMIC neurosurgeons are seldom included in the peer-reviewed review articles and clinical guidelines that dictate international standards of care^[^3,7,10^]^.

Various barriers are responsible for this inequality. Shortages of funding, limited infrastructure, and limited research mentorship are still major challenges^[^5,11^]^. Exorbitant publication charges, averaging $3000–$3500 per article, are also a major barrier for most LMIC authors^[^12^]^. Language barriers, limited access to journals, and the failure of protected research time foster cycles of low productivity and publication bias^[^3,11^]^. Consequently, the under-resourced world continues to be underrepresented in neurosurgical scholarship worldwide.

Closing these gaps necessitates intentional structural and collaborative action. Specialists call for minimized or waived publication charges, fair international collaborations, and focused investment in regional research capability^[^9,13,14^]^. There is evidence that extended partnership and committed funding have a substantial benefits to LMIC authorship and research productivity^[^9,14^]^. Narrative and systematic reviews also provide an entry point for LMIC researchers, which is less resource-demanding but high in academic value^[^15^]^.

This narrative review critically examines international imbalances in the neurosurgical literature, discusses major obstacles to inclusive authorship, and proposes a framework for a more equitable research environment. A narrative design is especially appropriate here to synthesize heterogeneous bibliometric results, address situated challenges, and incorporate policy considerations that a systematic review might miss, while emphasizing the necessity of concerted efforts toward worldwide neurosurgical equality^[^1,13,14,16^]^.

This manuscript is made compliant with the TITAN checklist to ensure transparency in the reporting of artificial intelligence^[^17^]^.

## Methods

This narrative review synthesized evidence on global disparities in neurosurgical literature. A structured search of PubMed, Scopus, and Google Scholar was conducted to identify articles published between January 2010 and August 2025. Search terms included combinations of “global neurosurgery”, “neurosurgical literature”, “bibliometric analysis”, “authorship disparities”, “low- and middle-income countries”, “high-income countries”, “open access publishing”, and “research equity”. Additional references were retrieved through manual screening of bibliographies and relevant professional reports.

Articles were eligible if they examined neurosurgical research output, authorship trends, publication barriers, or equity initiatives at a regional or global level. Bibliometric studies, reviews, commentaries, and perspective papers were included. This narrative review did not undertake any formal risk of bias or quality appraisal. Instead, emphasis was placed on mapping bibliometric trends, identifying structural barriers, and synthesizing proposed strategies to advance equitable representation in neurosurgical scholarship. A detailed overview of the databases searched, search parameters, eligibility criteria, and analytical approach is provided in Supplemental Digital Content Table 1, available at: http://links.lww.com/MS9/B213.

HIGHLIGHTS

- Global neurosurgical literature is dominated by high-income countries.
- Low- and middle-income countries (LMICs) face major barriers, including high article processing charges, limited access, and weak infrastructure.
- LMIC authors remain underrepresented in first and senior authorship roles.
- Regional case studies reveal shared gaps in workforce, training, and research capacity.
- Targeted collaborations and open-access initiatives improve neurosurgical equity.

### Bibliometric trends

Over the past decade, the global output of neurosurgical review publications has expanded considerably; however, this growth has been disproportionately driven by high-income countries (HICs). Bibliometric analyses demonstrate that more than two-thirds of reviews originate from North America and Western Europe, with the United States, the United Kingdom, and Germany consistently ranking as the top contributors^[^18,19^]^. By contrast, LMICs, including large regions such as sub-Saharan Africa, South Asia, and Latin America, account for only a small fraction of review publications despite carrying a substantial share of the global neurosurgical disease burden^[^20–22^]^.

This imbalance is further highlighted when outputs are examined by WHO region. While the Region of the Americas and the European Region contribute the majority of publications, the African and South-East Asian Regions together contribute fewer than 5–10% of indexed neurosurgical reviews^[^19,21^]^. Such underrepresentation is not merely a matter of research productivity but reflects systemic barriers: limited funding for academic work, lack of dedicated research infrastructure, and restricted access to international journals^[^20,23^]^. This bibliometric pattern reflects a structural inequity in which clinical realities in LMICs are rarely translated into consolidated evidence through reviews, thereby perpetuating invisibility in the global neurosurgical discourse.

**Figure 1.**
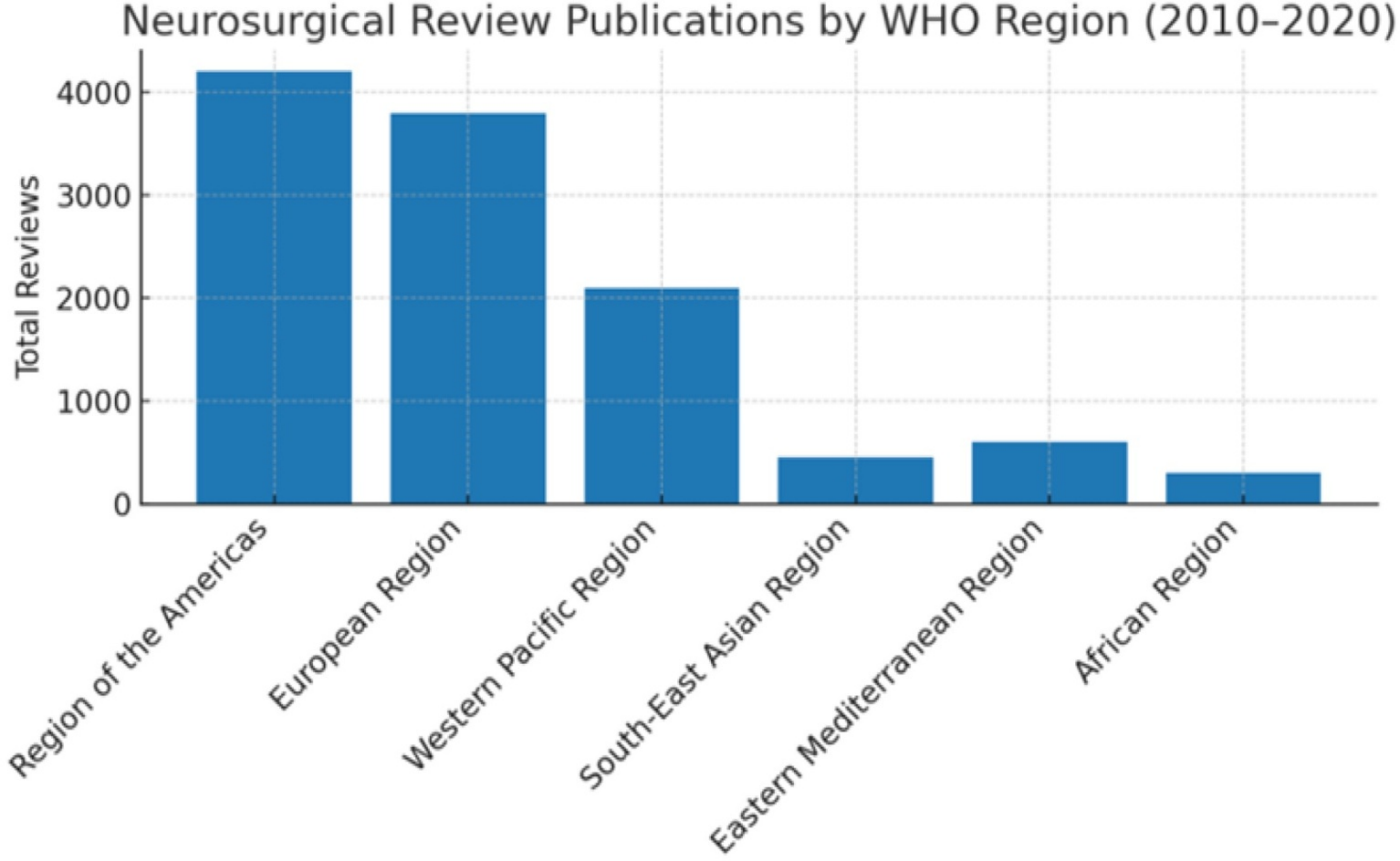
Global distribution of neurosurgical review publication output, illustrating regional disparities and the predominance of contributions from high-income countries compared with low- and middle-income regions.

**Interpretive synthesis**: The bibliometric patterns present a self-reinforcing cycle where research output increases visibility, visibility attracts funding, and funding further strengthens publication outcomes. Breaking this cycle will require targeted structural reforms and equitable resource distribution. Table 1 summarizes neurosurgical review publication output across WHO regions, highlighting the dominance of high-income regions compared to low- and middle-income regions. The geographic distribution of neurosurgical review output is illustrated in Figure 1.

**Table 1:** Neurosurgical review publication output by WHO region (2010–2020).

WHO region	Total reviews (2010–2020)	% of global output	Top-contributing countries
Region of the Americas	4200	34	United States, Canada, Brazil
European Region	3800	31	United Kingdom, Germany, France
Western Pacific Region	2100	17	China, Japan, Australia
South-East Asian Region	450	4	India, Thailand, Bangladesh
Eastern Mediterranean Region	600	5	Turkey, Iran, Saudi Arabia
African Region	300	2.5	South Africa, Nigeria, Egypt

### Barrier to research publications in LMICs

Researchers in LMICs face significant funding constraints when seeking to publish neurosurgical review articles. Institutions rarely offer research support, and authors often encounter prohibitive article processing charges (APCs) – sometimes as high as USD 3300–on their own^[^5^]^. Conversely, several other journals that claim to encourage authors by waiving APCs for publication secretly impose large fees disguised as editing service charges. Although minimal for larger institutions, these charges represent a major barrier for smaller LMIC centers, silencing valuable research that could advance neurosurgical knowledge and improve patient care worldwide^[^24^]^. Consequently, HICs contribute a major share of the neurosurgical literature^[^5^]^.

Such economic obstacles are also augmented by a lack of access to academic materials. In LMICs, scholars face a major challenge with the accessibility of scholarly literature, mainly because of the absence of institutional subscriptions to major databases and journals. Other programs, such as HINARI by WHO under Research4Life, aim to provide free or low-cost access to thousands of journals, yet many researchers, especially those in upper-middle-income countries, remain ineligible^[^25,26^]^. These challenges are not the end, but rather a beginning where collaborative efforts pave the way for meaningful progress. Unfortunately, LMIC authors are overrepresented as middle or supporting authors, up to 72%, while HIC partners are overwhelmingly assigned first- and corresponding-author roles. This contributes to “interpretive marginalization” and reinforces credibility imbalances^[^27^]^.

The institutional and national research infrastructure is also frequently insufficient in LMICs. Numerous academic centers operate in a setting where research culture is virtually non-existent. The much-needed requirements, including special funding, administrative backup, and technical facilities, are not always in sufficient supply. The internet connection, secure computing devices, and necessary tools, such as statistical software, are often unreliable or outdated. In spite of these restrictions, the academic staff is nevertheless still encumbered with the expectation of publishing prolifically. Career development hinges on producing multiple research publications, regardless of the institutional constraints. This dislocation exerts a huge burden on researchers, who have to overcome institutional obstacles in their attempts to meet unrealistic standards. What follows is burnout, undermined research, and unfair academic development^[^28^]^.

Furthermore, comparative evaluations of research output to disease burden reveal a significant disparity. For instance, in Africa, the PubFocus ratio is only one publication per unit of traumatic brain injury (TBI) burden. In contrast, the United States and Canada have a ratio of 91, indicating a significant disparity in the representation of research around the world. This mismatch reveals a more fundamental ethical motivation: the urgent need to expand neurosurgery research capacity in low-resource settings. This should include equitable possibilities for publishing^[^28^]^.

Breaking such obstacles requires monetary and institutional aid. Mechanisms of funding, such as research grants, journal fee waivers, and institutional support, are necessary. Systems-level interventions that foster research ability and confidence are also critical. The strong model used by programs such as AuthorAID provides a relationship between LMIC researchers and volunteer mentors. These mentors offer editorial advice, instruct on publication standards, and assist in manuscript reviews. These programs contribute to closing the publishing gap, allowing researchers to make a valuable contribution to the neurological world.

Table 2 summarizes the major barriers faced by LMIC authors, including APCs, limited infrastructure, and authorship inequity.

**Table 2:** Barriers to publication in LMICs.

Category	Barrier	Impact
Financial constraints	- High article processing charges (APCs) (up to USD 3,300)	- Limits authors’ ability to publish in open-access journals
	- Hidden charges disguised as “editing services”	- Adds unexpected cost burdens, especially for smaller institutions
Limited access to literature	- Lack of institutional subscriptions to key databases/journals	- Restricts the ability to conduct high-quality literature reviews
	- Ineligibility for programs like HINARI among many upper-middle-income countries	- Further limits access to free/low-cost resources
	- Poor infrastructure (internet, hardware) and lack of training	- Hinders effective use of available access programs
Authorship inequities	- LMIC authors are often relegated to middle/senior authorship in collaborations with HICs	- Reduces the visibility, recognition, and credibility of LMIC researchers
Editorial participation	- Limited participation in peer review due to clinical workload and lack of training	- Limits influence in shaping global neurosurgical literature
Institutional deficiencies	- Absence of research culture, funding, and administrative/technical support	- Hinders productivity despite pressure to publish
Infrastructure gaps	- Lack of access to statistical software, secure computers, and stable internet	- Limits the ability to conduct and submit quality reviews
Ethical imbalance	- Mismatch between disease burden and research output (e.g., Africa’s low PubFocus ratio vs HICs)	- Highlights global neurosurgical research inequity
Need for mentorship and support	- Scarcity of writing mentorship, editorial support, and academic English training	- Contributes to the publishing gap; limits manuscript acceptance
	- Initiatives like AuthorAID are underutilized or underpromoted	- Missed opportunity for capacity building and skills development

### Case studies from underrepresented regions

Despite LMICs bearing a much larger share of the global neurosurgical disease burden, most of the world’s neurosurgical training and published research still comes from HICs. Recent estimates show that, while nearly 73 000 neurosurgeons practice worldwide, their distribution is highly uneven. HICs have about 2.4 neurosurgeons per 100 000 people, compared to just 0.12 per 100 000 in low-income countries^[^29^]^. If you consider that more than 13 million people need neurological surgery each year, then 78% of them live in LMICs^[^21^]^.

**A) Africa**:

Workforce

Despite carrying one of the heaviest burdens of neurosurgical disease, Africa continues to face deep structural challenges. On average, there is only one neurosurgeon for every 3–6 million people, compared with about one for every 80 000 people in many HICs^[^21^]^.

Training and education: Most neurosurgical training opportunities in Africa are concentrated in a handful of major cities, leaving large regions without access to specialized education. Young neurosurgeons often describe the difficulty of advancing their skills, and around 90% lack cadaver dissection labs; over half have never attended an international neurosurgical conference, and two-thirds have minimal exposure to research opportunities. These gaps not only limit professional growth but also reinforce the continent’s low visibility in global neurosurgical literature^[^30^]^.

#### Research and publication

In Africa, there is a tremendous burden of neurological disease due to traumatic brain injuries after road accidents, which also includes children living with untreated hydrocephalus and ongoing challenges from neurological infectious conditions. Yet, when it comes to the global research stage, the continent’s voice is barely heard. African authors contribute less than 2% of all neurosurgical publications worldwide, and most of these are small case reports or single-center series rather than the large clinical trials or systematic reviews that shape international guidelines. This imbalance highlights the disconnect between the region’s disease burden and its visibility in the scientific literature^[^7,31^]^.

**B) South Asia:**

**Workforce:**

In recent years, South Asia has demonstrated remarkable expansion in neurosurgery. Countries such as India, Pakistan, Bangladesh, and Nepal are gradually increasing their neurosurgical workforce.

#### Training and education

The region has experienced a progressive expansion in neurosurgical training capacity and stronger academic participation. Recent work from Nepal demonstrates a rise in neurosurgery-related publications over the past decade^[^32^]^. Even with an increase in training programs and a rising number of publications, South Asian research remains with low visibility in global reviews and guidelines. A bibliometric analysis of the South Asian part of the broader region shows that most of their publications are still case reports or series, and in many cases, progress in research seems to depend mainly on external funding or international collaborations rather than local, sustainable structures^[^9^]^.

**C) Latin America**:

The neurosurgical workforce in Latin America has expanded over the past few years. The majority of neurosurgeons are concentrated in urban and highly developed regions, while rural and less-developed regions continue to face critical shortages. Neurosurgical training opportunities and academic collaborations have increased across Latin America, although the quality of residency programs and research exposure remains highly variable. However, the number of neurosurgical publications from Latin America has increased. Research activity remains largely centralized, with the majority of contributions originating from a limited number of countries and published primarily in regional journals that receive limited global attention. A survey conducted by the Federación Latinoamericana de Sociedades de Neurocirugía highlighted significant needs in structured residency programs, limited opportunities for exchange rotations, and uneven access to research platforms^[^33^]^.

Across Africa, South Asia, and Latin America, significant advancements have been made toward expanding neurosurgical capacity through regional collaborations and improved training programs. Despite these advancements, substantial disparities persist. In Africa, the severe shortage of trained neurosurgeons, limited access to cadaveric laboratories, and scarce research infrastructure continue to hinder growth. South Asia has experienced a rise in training opportunities and academic engagement, yet much of its research output remains dependent on external funding and collaborations. In Latin America, established professional bodies and regional cooperation have supported structured training initiatives, but research activity remains concentrated in a few countries and institutions. Common to all three regions are the challenges of uneven workforce distribution, inadequate infrastructure for research, and underrepresentation in global neurosurgical publications. Strengthening local capacity, ensuring equitable access to education, and fostering regionally led research networks are essential steps to bridge the gap between disease burden and academic contribution worldwide.

**Figure 2.**
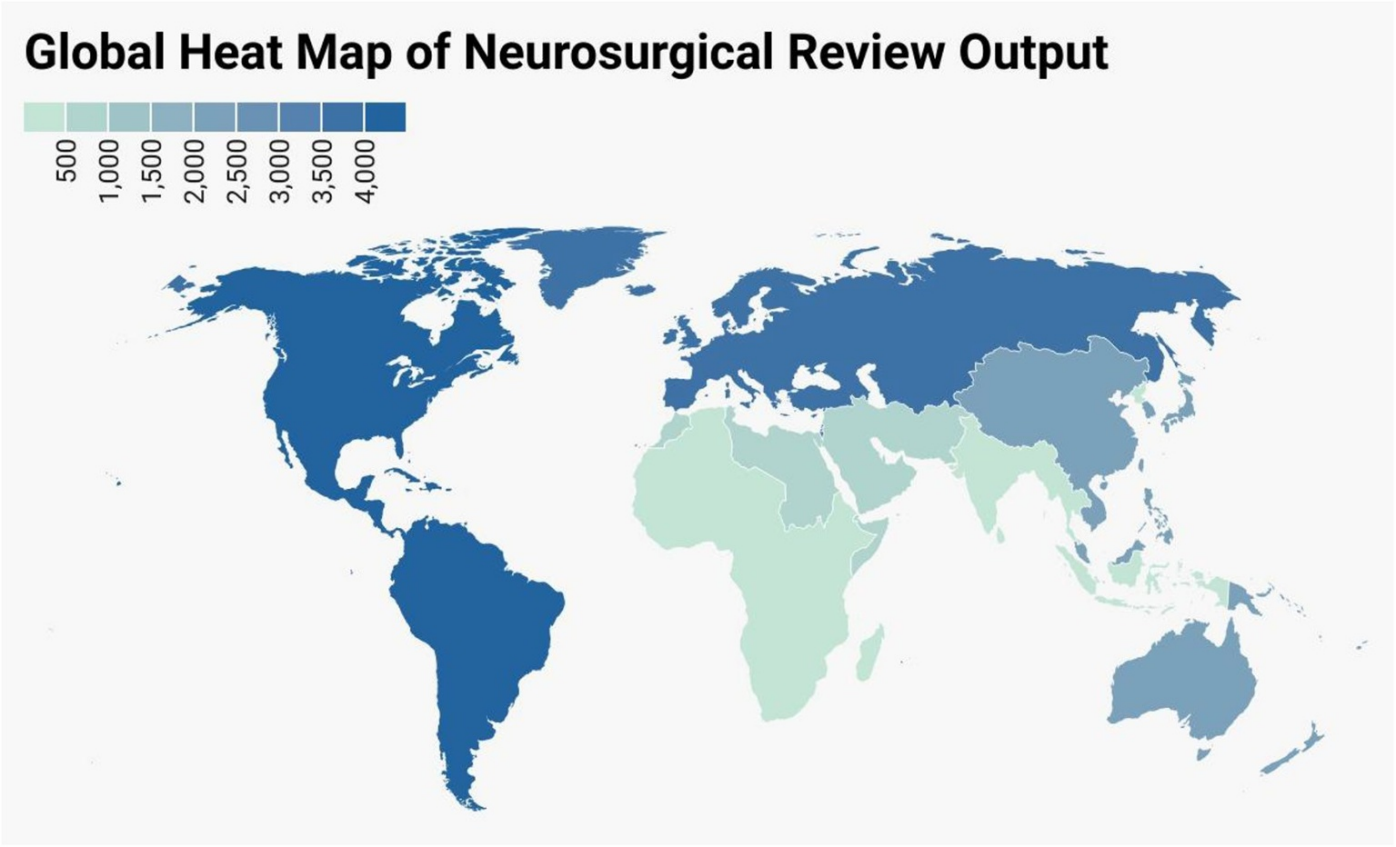
Global heat map of neurosurgical review output.

Figure 2 depicts a global heat map of neurosurgical review output, highlighting severe underrepresentation in LMIC regions.

#### Consequences of literature disparities

A) Skewed guidelines and external validity gaps:

The lack of data from LMICs in neurosurgical research weakens the external validity and global relevance of clinical guidelines. When most evidence comes from wealthy nations, treatment protocols overlook the realities faced in low-resource settings. This can widen the gaps in outcomes around the world.

The Global Neurotrauma Outcomes Study revealed significant gaps in emergency neurosurgical care for TBI. Patients in lower-income countries have different case profiles. They also deal with limited access to monitoring and surgery, which leads to higher mortality rates^[^34^]^. In Tanzania, the 2-week death rate for severe TBI was 67% at a major tertiary hospital. This rate is much higher than what is usually reported in HICs^[^35^]^. These discrepancies show how guidelines based on HIC data can misrepresent the standards that can realistically be met in LMIC contexts.

Access to intensive care, including ICP monitoring and admission to neurocritical care, varies widely between HICs and LMICs. These differences in availability directly influence how patients are prioritized and can significantly affect outcomes^[^36^]^. When guidelines rely primarily on evidence from HICs, they often overlook the realities in low- and middle-income areas. Without adjustments, these guidelines can be hard to follow and may unintentionally widen gaps in care quality and survival^[^37^]^.

B) Under recognition of region-specific burdens and phenotypes:

The focus on HIC literature misses the neurosurgical issues unique to LMICs. This gap skews our view of global disease patterns. By ignoring these data, we overlook local challenges and weaken the global understanding needed to develop effective prevention and treatment strategies.

Most of the studies come from HICs. They often miss the injury patterns that are important in low- and middle-income areas. In Pakistan, a national survey found that road traffic collisions are the leading cause of TBI^[^38^]^. In Ethiopia, a study shows that patients with TBI often reach the hospital late, and their hospital death rate is much higher than what is typically reported in HICs^[^39^]^. Regional studies have examined how often traumatic head injuries occur and what risk factors play a role. They also reveal issues in systems that lead to worse outcomes. These findings highlight the importance of strategies for prevention and management, which reviews that concentrate on HICs often miss^[^40^]^.

Infectious neurosurgical diseases often get ignored. Neurocysticercosis is a major cause of acquired epilepsy in LMICs where it is prevalent. Recent data from Zambia show that point-of-care tests can help identify and manage this condition more effectively^[^41^]^. Yet, these advances rarely appear in guidelines for HICs, where neurocysticercosis receives little attention. In sub-Saharan Africa, post-infectious and TB-related hydrocephalus put a heavy load on pediatric neurosurgery. Regional studies show high caseloads and specific causes^[^42^]^. In children with hydrocephalus from TB meningitis, HIV significantly raises the risk of death in the hospital^[^43^]^. Randomized trial data show that surgically treating infants with post-infectious hydrocephalus can lead to long-lasting improvements in brain growth and neurodevelopment^[^44^]^. Spinal tuberculosis still leads to major disability in LMICs. However, reviews often overlook local data on functional outcomes. This information is important for planning rehabilitation and determining when surgery is necessary^[^45^]^. These omissions slow down the development of fair global strategies that address the various neurosurgical diseases.

C) Missed innovation from resource-constrained environments:

The limited representation from LMICs in neurosurgical reviews leads to missed chances for innovation based on local needs. Areas with few resources often drive the creation of affordable and scalable solutions. These solutions could influence global best practices, but their input is mostly ignored in mainstream literature.

The lack of representation for research from LMICs means that important local innovations often do not reach the global community. Randomized and long-term studies from Africa on infant post-infectious hydrocephalus have improved how doctors select and follow up with patients after endoscopic or shunt procedures. These findings could help similar cases around the world, but they are slow to be included in global reviews^[^44,46^]^. Registries and provincial studies of pediatric hydrocephalus in sub-Saharan Africa provide valuable insights. They point out predictors of complications and suggest methods to organize services. This information can help in creating scalable and locally suitable care pathways^[^43^]^.

In LMICs, researchers are creating neurotrauma registries and care bundles for patients from the roadside to the ICU. However, since this data is not often included in global reviews, the progress in developing practical, tiered approaches to TBI care is slow^[^47^]^. The limited use of these findings in global reviews further slows the spread of practical, tiered models of care. These models could help neurosurgical systems worldwide.

Literature gaps not only sustain inequality but also hide valuable knowledge from areas where innovation is needed. A global neurosurgical evidence base must recognize and include these contributions. This is essential to ensuring that improvements in care reflect the diversity of real-world clinical situations.

#### Opportunities and initiatives to bridge gaps

Global disparities persist in neurosurgical research, with LMICs underrepresented in publications despite housing 80% of the world’s population. Limited funding, outdated infrastructure, and lack of mentorship contribute to low academic output and underrepresentation in international collaborations^[^48–50^]^. Gender and racial inequities further compound these disparities, as male principal investigators receive disproportionately higher funding, and LMIC authors are often middle authors rather than first or last authors^[^51,52^]^. Technological gaps also affect research opportunities and access to neurosurgical education^[^53^]^.

A bibliometric analysis of 337 global neurosurgery publications (2011–2021) revealed the dominance of HIC authors: 41.1% from the US, 4% from Canada, and 3.9% from the UK, while LMIC authors were most commonly from Uganda (4.2%), Tanzania (2.6%), Cameroon (1.8%), and India (1.8%). Over 28% of publications lacked any LMIC authors, and only 11% had three or more^[^50^]^.

Country-level analyses further illustrate LMIC challenges. Hoz *et al*^[^54^]^ examined PubMed-indexed research productivity of Iraq-based neurosurgeons from 2003 to 2021. Only 52 papers were published, clustered between 2012 and 2021, with most originating from Baghdad (*n* = 46) and the Neurosurgery Teaching Hospital in Baghdad contributing 38 publications. Despite a promising growth trend from 2017 to 2021 (average 12 publications/year), peripheral regions remained underrepresented, highlighting the need for political, financial, and system-level strategic planning to sustain research growth^[^54^]^. These studies underscore the need for expanded research platforms, mentorship, and collaborative networks to improve LMIC representation^[^55^]^.

Medical students are also emerging as key contributors. Safa *et al*^[^56^]^ showed that Mission: BRAIN’s European chapters expanded by 150% in 2 years, doubling membership, increasing educational events by 325%, and fostering collaboration among students and healthcare providers. Members reported heightened awareness of global neurosurgical needs and active engagement in outreach, with 59% indicating contributions to reducing disparities^[^56^]^. Varela *et al*^[^57^]^ further demonstrated that a 2-year intensive research initiative could rapidly increase scholarly output in departments without residents. Medical student-led publications rose by 1000%, pre-residency fellow publications by 4900%, and overall learner participation from 31 to 72%^[^57^]^. This confirms that structured mentorship and early student involvement are highly effective, reproducible, and scalable strategies for increasing academic productivity and promoting equity.

#### Open-access and inclusive publication model

Open-access publishing offers an invaluable means of disseminating neurosurgical knowledge without paywalls. Yet, alarming disparities persist: LMIC researchers account for less than 8% of publications in neurosurgery’s open-access journals over the past 5 years^[^5,24^]^. One bibliometric study found that countries in the low- and lower-middle-income brackets contributed less than 8% of the output in 18 dedicated OA neurosurgical journals^[^58^]^. The study discussed how this disparity reflects systemic limitations in research infrastructure, funding, and training in LMICs, despite these regions carrying a disproportionately high neurosurgical disease burden. It noted that the imbalance in authorship risks skewing the global evidence base toward high-income contexts, where disease profiles and available resources differ, thereby limiting the applicability of published findings to LMIC settings. The authors further emphasized that structural barriers such as high APCs, limited mentorship, and lower neurosurgeon density continue to hinder LMIC contributions, even in OA platforms designed to promote wider participation until 2023^[^58^]^. APCs still continue to be a huge systemic barrier. Among 53 neurosurgical journals surveyed, the median APC was USD 3286, with some reaching as high as USD 7827, placing OA publishing beyond reach for many LMIC authors^[^59^]^. Even when journals claim to support authors, hidden “editing service” fees can disproportionately burden LMIC researchers.

Despite these challenges, some publishers do offer structured waivers. World Neurosurgery grants automatic 100% APC waivers to authors whose affiliations are entirely within low-income economies, and 50% reductions under specific financial constraints for others. Springer Nature provides similar policies, with full APC waivers for authors from low-income countries and 50% discounts for many lower-middle–income nations, covering more than €18 million in APCs^[^60^]^. Moreover, Nature Research journals launched a 2023 initiative that automatically provides Gold OA at no cost for accepted papers with corresponding authors from over 70 LMICs^[^61^]^.

Global initiatives further support equity in access. The WHO-led HINARI program, part of Research4Life, provides free or low-cost access to over 8,500 biomedical and health journals for eligible LMIC institutions, bridging critical access gaps^[^62^]^. However, critics argue that even tiered waiver models often fail to reach upper-middle–income countries or researchers without institutional backing, limiting their overall impact^[^63^]^. Nevertheless, evidence suggests OA can improve equity. In Tanzania, 62% of neurosurgical articles authored by local researchers were published open access, and OA status significantly increased the likelihood of Tanzanian authors being listed as first or last authors^[^64^]^.

#### Regional collaborations, South–South partnerships, and student involvement

Regional and South–South collaborations are proving pivotal in fostering LMIC academic leadership and autonomy. A scoping review of global neurosurgical outreach revealed that 28.4% of HICs collaborated with 63.4% of LMICs, with many initiatives involving countries such as Uganda, Tanzania, and Kenya^[^65^]^. These partnerships, typically involving training and research, highlight the importance of sustained engagement that translates into local capacity building rather than one-off interventions.

The African Neurosurgical Research Collaborative exemplifies this model, enabling multicenter studies across more than 20 African nations on topics such as TBI and pediatric hydrocephalus^[^66^]^. Such collaborations empower LMIC authors to lead projects, reducing reliance on HIC investigators. Paradie *et al*^[^50^]^ further demonstrated that long-term HIC–LMIC partnerships strengthen LMIC authors’ independence, though regional networks remain the most effective at building sustainable autonomy.

Government-supported models also show strong potential. The Egyptian Knowledge Bank, a national initiative in partnership with Springer Nature and Elsevier, provides free access to international journals and supports local open-access publishing. Journals such as the Egyptian Journal of Neurosurgery offer no-cost publishing to local researchers, directly removing APC barriers and fostering wider participation^[^67^]^. Student-led initiatives further drive inclusivity. Mission: BRAIN and similar organizations mobilize medical students to engage in research design, data collection, and dissemination^[^56^]^. Varela *et al*^[^57^]^ confirmed that structured student-led research initiatives can generate substantial scholarly output, independent of residency training programs.

To strengthen these efforts, journals should expand fee waivers and mentorship programs for LMIC authors, global neurosurgery societies should facilitate South–South research consortia and open data platforms, and policymakers should invest in regional research infrastructure and equitable publishing access. Together, these coordinated actions can sustain authorship equity and advance global neurosurgical scholarship^[^57^]^.

Table 3 outlines key global programs, including AuthorAID, Mission: BRAIN, and AFNRC, which contribute to research equity.

**Table 3:** Examples of initiatives to address global neurosurgical publication disparities.

Initiative/approach	Description and impact	Citations
Mission: BRAIN Foundation	Global chapters combining care, education, and research training for LMICs	Safa *et al* ^[^55^]^
Global Champions Program	Free English editing, reducing linguistic barriers, and increasing LMIC author acceptance	Shlobin *et al* ^[^68^]^
HIC–LMIC Collaborations	Joint research, mentorship, and funding, enhancing LMIC author independence	Cabrera-Mendoza *et al* ^[^69^]^
African Neurosurgical Research Collaborative (AFNRC)	Multicenter studies across >20 African countries shaping regional guidelines	Piquer *et al* ^[^65^]^
WFNS Young Neurosurgeons Forum	Mentorship pairing LMIC neurosurgeons with global mentors	Safa *et al* ^[^55^]^
PGSSC Mentorship Program	HIC–LMIC mentorship in health systems and neurosurgery policy, producing co-authored publications	Cabrera-Mendoza *et al* ^[^69^]^

#### Policy and structural recommendations

Tackling the disparities in neurosurgical review publications requires multi-level collaboration among journals, professional societies, funding agencies, and policymakers.

At the editorial level, diversification of journal leadership and reviewer pools is essential. Increasing representation from LMICs on editorial boards brings contextual expertise and promotes inclusivity in peer review decisions^[^68,70^]^. Journals should adopt equitable peer review policies that recognize barriers faced by LMIC authors, such as limited access to advanced statistical or language editing support, while maintaining scientific standards^[^71^]^.

Financial barriers also need to be addressed. Implementing waivers or tiered article processing charges for authors from LMICs, alongside stronger open-access models, can reduce exclusion based on funding capacity^[^69,72^]^. Concurrently, funding agencies and global health organizations should prioritize capacity-building and mentorship programs directed at neurosurgical research in LMICs through dedicated grants and regional research networks^[^73^]^.

Moreover, professional societies and global neurosurgery networks, for instance, the World Federation of Neurosurgical Societies Global Neurosurgery Committee, should advocate for global neurosurgery as a formal academic and policy subspecialty^[^73^]^. Embedding this agenda in national and international frameworks can help create a policy logic: barriers → disparities → interventions → measurable outcomes, underlining that equitable research output is an intentional outcome of structural reform rather than an incidental byproduct.

#### Call to action

- Journals: Diversify editorial boards and implement equity-based peer-review practices.
- Funders: Allocate grants and mentorship specifically for LMIC-led neurosurgical research.
- Societies: Endorse global neurosurgery as a defined subspecialty with dedicated leadership.
- Policymakers: Integrate research equity into national/regional health and academic policy frameworks.

**Figure 3.**
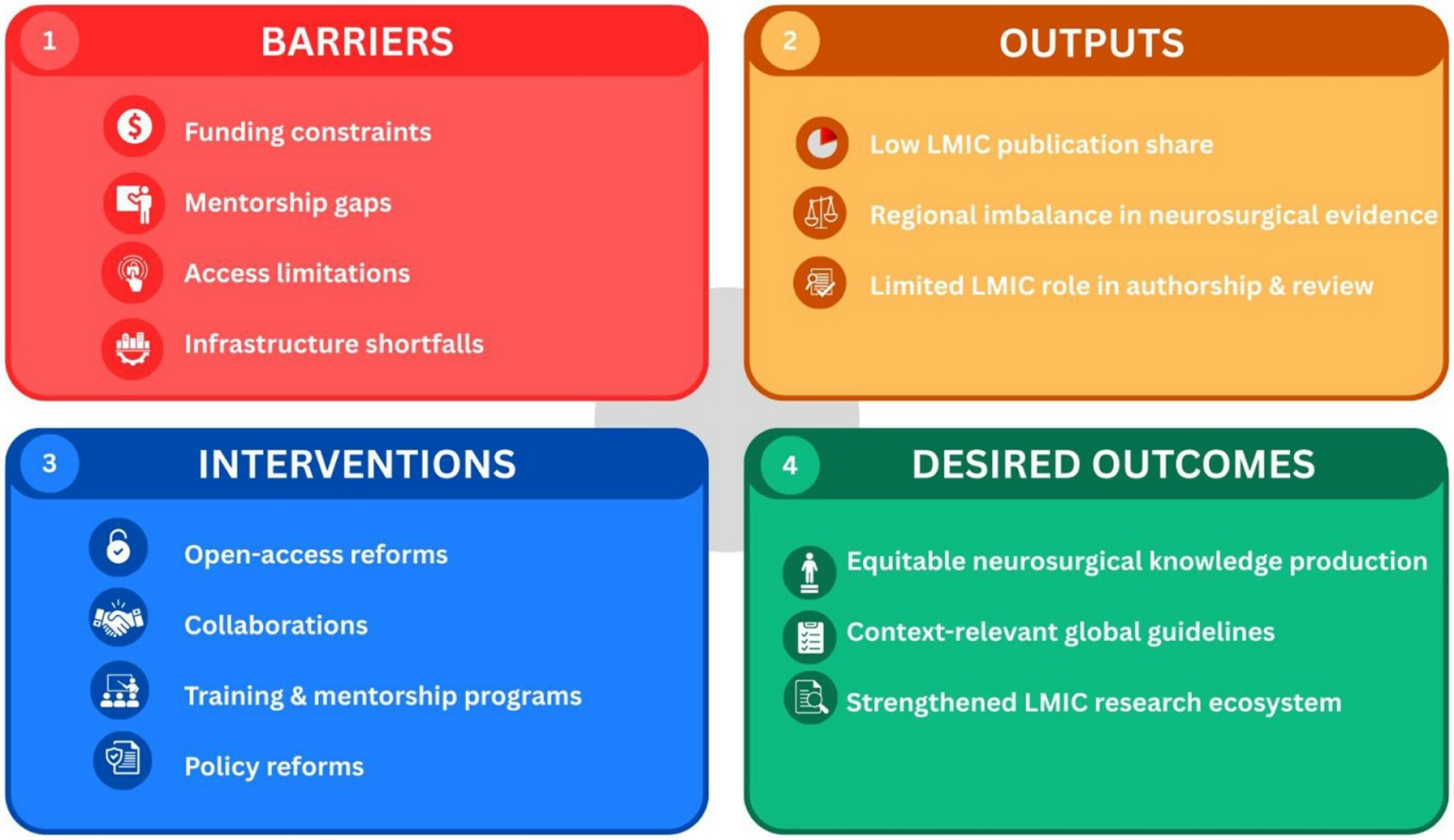
Conceptual framework: Barriers → Disparities → Interventions → Outcomes.

Figure 3 provides a conceptual framework linking publication inequities to actionable solutions.

## Conclusion

This narrative review highlights the persistent global disparities in neurosurgical research output. HICs continue to dominate the literature, while contributions from LMICs remain limited. Barriers such as resource constraints, lack of infrastructure, limited training opportunities, and challenges in research funding have contributed to these inequities. Although collaborative efforts and initiatives to strengthen global neurosurgical capacity are increasing, substantial gaps remain. Addressing these disparities will require sustained international partnerships, improved access to funding, mentorship, and capacity-building programs. Bridging these divides is essential to ensuring that neurosurgical research reflects global disease burdens and contributes equitably to advancements in patient care worldwide.

## Data Availability

All data used in this narrative review are publicly available and sourced from previously published studies. No new data were generated for this work. All included articles have been appropriately cited within the manuscript and are available through the references section.

## Research registration unique identification number (UIN)

The research registration unique identification number (UIN) was not required because it is a narrative review.
